# Anti-HMGCR Specificity of HALIP: A Confirmatory Study

**DOI:** 10.1155/2020/6292631

**Published:** 2020-07-16

**Authors:** Andrés Baucells, Maria Angeles Martínez, Marcelo Alvarado-Cardenas, Anaís Mariscal, Laura Martinez-Martinez, Cándido Juárez, Albert Selva-O'Callaghan

**Affiliations:** ^1^Immunology Department, Hospital de la Santa Creu i Sant Pau, Barcelona 08041, Spain; ^2^Systemic Autoimmune Diseases Unit, Hospital Vall d'Hebrón, Medicine Department, Universidad Autónoma de Barcelona, Barcelona, Spain

## Abstract

A distinctive new indirect immunofluorescence pattern in liver tissue has been associated with anti-HMGCR autoantibodies. It is known as HALIP (HMGCR Associated Liver Immunofluorescence Pattern). In this study, we furthered the original studies to demonstrate the association of anti-HMGCR antibodies with the HALIP. Human anti-HMGCR antibodies from patients' sera were purified and incubated with rat triple tissue (kidney/stomach/liver). A characteristic HALIP was observed. Additionally, a colocalization assay of human anti-HMGCR antibodies with rabbit polyclonal anti-HMGCR antibodies showed colocalization of both immunofluorescence patterns. This study confirms that the HALIP is due to human anti-HMGCR antibodies.

## 1. Introduction

Idiopathic inflammatory myopathies (IIM), also known as myositis, are a group of heterogeneous disorders characterized by a distinct inflammatory infiltrate in muscle tissue [1]. Several phenotypes linked to specific autoantibodies have been described [1, 2]. One of these phenotypes includes patients exposed to statins who develop a statin-associated autoimmune myopathy with antibodies against the pharmacologic target of statins, the 3-hydroxy-3-methylglutaryl-coenzyme A reductase (HMGCR). These autoantibodies and their link to a specific myositis phenotype were described in 2010 by Mammen et al. [3]. The pathological characteristics of this statin autoimmune myopathy are those of the immune-mediated necrotizing myopathy (IMNM), defined by the presence of necrotic fibres found in muscle biopsy. Severe proximal muscle weakness and high levels of creatinine kinase are also hallmarks of the disease [4, 5].

In 2016, Alvarado-Cardenas et al. [6] described a distinctive, novel indirect immunofluorescence (IIF) pattern on rat liver sections associated with anti-HMGCR antibodies. They called it the HALIP (HMGCR Associated Liver IFL Pattern). Here, we further identify these autoantibodies as responsible for the previously described characteristic IIF pattern. Our findings demonstrate that the HALIP is specific for human anti-HMGCR antibodies, as shown by the IIF pattern of the purified human anti-HMGCR antibodies and by the colocalization of these human autoantibodies with rabbit polyclonal anti-HMGCR antibodies.

## 2. Material and Methods

### 2.1. Material

#### 2.1.1. Sera

The human sera used in the assay were obtained from 3 patients with statin-associated necrotizing myopathy that tested positive with a high titre of anti-HMGCR antibodies by ELISA (INOVA Diagnostics, San Diego, CA). Also, three healthy human sera were used as negative controls. The diagnostic criteria for IMNM were based on the IMNM diagnostic and classification criteria proposed by the Muscle Study Group/European NeuroMuscular Centre (MSG/ENMC) [7].

#### 2.1.2. IIF Assays

All the IIF assays were performed on rat triple tissue INOVA slides according to the instructions of the NOVA Lite Rat Liver, Kidney, Stomach Kit (INOVA Diagnostics, San Diego, CA).

### 2.2. Purification of Human Anti-HMGCR Antibodies

5 *μ*g of recombinant HMGCR protein (Origene, Rockville, MD) was run on SDS-gel electrophoresis and transferred into a nitrocellulose membrane as previously described [8]. Briefly, we blocked the membrane with 5% casein in PBS and then cut two vertical strips at the ends of the membrane. The strips were then incubated with the human anti-HMGCR serum and, following the blot protocol [8], the position of the protein was localized. Next, we incubated the remaining section of the membrane overnight, covered with 6 ml of anti-HMGCR patient serum dilution (diluted 1/20 in 3% casein in PBS). We then washed the membrane three times with PBS 0.01% tween and cut a horizontal strip off the membrane horizontally 0.3 cm above and 0.3 cm below the localized protein. On continuation, we cut the strip into little pieces and placed them in a tube.

At this point, we eluted the antibodies bound to the pieces with 250 *μ*l of glycine pH = 2, for 2 minutes under agitation. The elute was then buffered with 25 *μ*l of Tris pH = 9.5 to take it back to pH = 7. Finally, we incubated 50 *μ*l of the buffered elute in rat tissue following the IIF INOVA kit instructions.

A strip was cut from the remaining part of the membrane and incubated with the HMGCR antibodies following the same protocol described above. It was used as a purification negative control.

### 2.3. Colocalization of Human and Rabbit Polyclonal Anti-HMGCR Antibodies

First, we incubated the rat tissue with 50 *μ*l of anti-HMGCR Rabbit Polyclonal Antibody (1/50 dilution in PBS) (Invitrogen, Frederick, MD) for 1 h in a humid chamber. After washing thrice with PBS, the tissue was incubated with goat anti-rabbit IgG (H+L) labelled with Alexa Fluor 594 (Invitrogen, Frederick, MD) for 1 h. We then incubated the tissue with the anti-HMGCR-positive human serum (1/80 in PBS) for 30 min, followed by washing and incubation with a drop of goat anti-human FITC IgG H&L conjugate (from the triple tissue INOVA kit) for another 30 min.

Each of the conjugates was tested individually to ensure there was no crosspositivity between the excitability wavelength of the two different fluorochromes.

### 2.4. Absorption of the Anti-HMGCR Antibodies

We incubated the serum of a patient with anti-HMGCR antibodies (1/80 dilution in PBS) with an excess of HMGCR recombinant protein (Sigma-Aldrich, St Louis, MO) at a concentration of 25 *μ*g/ml for 30 minutes. The absorbed serum was then poured on rat sections to test for the HALIP.

## 3. Results

### 3.1. Immunofluorescence Pattern of the Purified Human Anti-HMGCR Antibodies

**Figure 1:**
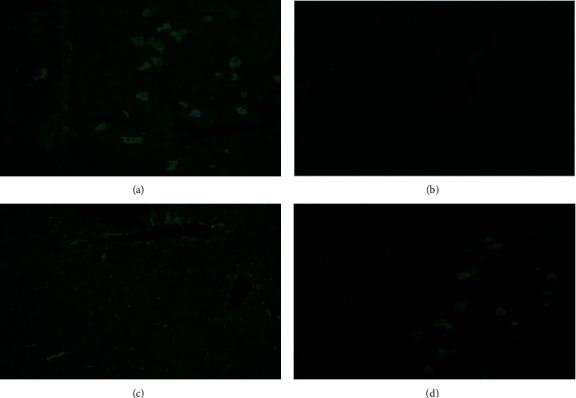
The figures portray a representative image of one of the anti-HMGCR human sera staining patterns on rat liver. (a) HALIP as described, staining some isolated hepatocytes on liver rat sections after incubation with an anti-HMGCR-positive human serum (dilution 1/80). (b) Normal human serum as negative control (dilution 1/80). (c) Anti-HMGCR-positive serum after absorption with HMGCR recombinant protein (dilution 1/80). (d) HALIP staining pattern after incubation with antibodies eluted from gel-fractionated recombinant HMGCR protein.

The anti-HMGCR patients' positive sera stain isolated hepatocytes located periportally on rat liver, as reported for the HALIP (Figure 1(a)). Rat triple tissue was also incubated with normal human sera as a negative control (Figure 1(b)). As shown in Figure 1(c), the HALIP disappeared after absorption of anti-HMGCR autoantibodies with recombinant HMGCR, supporting that specific anti-HMGCR antibodies are responsible for the pattern. This conclusion is further confirmed by the studies performed with purified antibodies. In fact, as observed in Figure 1(d), the IIF pattern on rat liver tissue of the purified anti-HMGCR antibodies reproduces the same pattern of the anti-HMGCR patients' positive sera on Figure 1(a). The purified negative control showed no staining on triple tissue (data not shown), thus confirming that the purification of the HMGCR autoantibodies is specific.

### 3.2. Immunofluorescence Pattern of the Colocalization Assay

**Figure 2:**
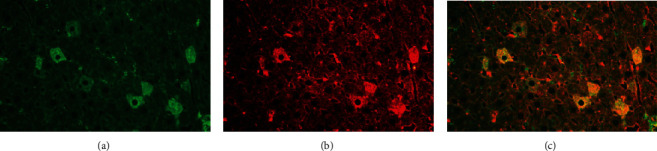
HALIP of (a) a human anti-HMGCR-positive sera conjugated with FITC, (b) polyclonal rabbit anti-HMGCR sera conjugated with Alexa 594, and (c) the merging of (a) and (b).

The results obtained above indicate that the differential expression of HMGCR in rat liver cells is responsible for the HALIP. A commercial antibody obtained by immunization with HMGCR would therefore reproduce the HALIP, recognizing the same hepatocytes as those in patients' sera. To demonstrate this, we incubated the rat triple tissue with both human and commercially obtained rabbit anti-HMGCR antibodies. As expected, we observed that the human anti-HMGCR antibodies (Figure 2(a)) stain exactly the same rat liver cells as the rabbit polyclonal anti-HMGCR antibodies (Figure 2(b)). This is clearly shown in the merged image (Figure 2(c)) that confirms the correspondence of the two stainings.

## 4. Discussion

In this study, using purified human anti-HMGCR antibodies and colocalization studies, we demonstrate that the HALIP is due to anti-HMGCR antibodies.

The fact that purified human anti-HMGCR antibodies are directed against some rat liver cells is not surprising because hepatocytes are responsible for the synthesis of 60-70% of the cholesterol for the whole body [9]. HMGCR is mainly expressed in periportal hepatocytes, as reflected by the HALIP. This distribution was previously reported in studies that analysed the effects of statin on the liver [10, 11]. These studies show that the initial expression of HMGCR in periportal hepatocytes changes drastically to a more generalized expression in rat liver after statin treatment. As rat and human HMGCR share 93% homology, the rat triple tissue is a good substratum to test for human anti-HMGCR autoantibodies [6]. Though HMGCR is present in the liver, anti-HMGCR antibodies are associated with myositis. To our knowledge, only one previous case of autoimmune hepatitis and anti-HMGCR statin-associated autoimmune myositis patient has been reported to date [12, 13].

As IIF is a common and inexpensive screening technique available in most clinical laboratories, the HALIP can be extremely useful for laboratory analysis. Rat triple tissue could be used as an initial screening test for anti-HMGCR antibodies in laboratories where the specific antigen detection assay is not available. Moreover, if a HALIP is observed in our routine work as an unexpected finding, the presence of anti-HMGCR antibodies should be tested.

Despite the specificity of the HALIP, confirmation with an antigen detection assay is strongly recommended, as we have observed similar patterns that were negative to anti-HMGCR antibodies in the confirmation test. Furthermore, a negative HALIP does not rule out the presence of anti-HMGCR antibodies, because the HALIP was not found in 2/23 anti-HMGCR-positive myositis patients, according to Alvarado-Cardenas et al. [6]. The reason for this discordance remains to be assessed.

In conclusion, our findings in this report confirm that anti-HMGCR antibodies are responsible for the HALIP on rat liver.

## Data Availability

The data used to support the findings of this study are included within the article.
